# Physiological markers of Trauma‐related nightmares among military personnel suffering from PTSD: A multicenter home‐recording study

**DOI:** 10.1111/pcn.70038

**Published:** 2026-02-14

**Authors:** Dorone Feingold, Michael Quiquempoix, Marion Remadi, Gilles Sipahimalani, Bertrand Lahutte, Damien Léger, Danielle Gomez‐Merino, Mounir Chennaoui, Emeric Saguin

**Affiliations:** ^1^ Université Paris Cité UMR 7330 VIFASOM (Vigilance Fatigue Sleep and Public Health) Paris France; ^2^ French Armed Forces Biomedical Research Institute (IRBA) Bretigny‐sur‐Orge France; ^3^ Department of Psychiatry Begin National Military Teaching Hospital Saint‐Mandé France; ^4^ Department of Psychiatry Percy National Military Teaching Hospital Clamart France; ^5^ APHP, Hôtel Dieu, Centre du Sommeil et de la Vigilance CRPPE Sommeil Vigilance et Travail Paris France

**Keywords:** biomarkers, military personnel, nightmares, PTSD, sleep

## Abstract

**Aim:**

Despite being one of the most disabling symptoms of post‐traumatic stress disorder (PTSD)—disrupting sleep continuity, reinforcing hyperarousal, and worsening psychiatric comorbidity—the physiological signature of Trauma‐related nightmares (TRNs) under naturalistic sleep conditions remains poorly characterized.

**Methods:**

We used home‐based, multi‐sensor devices from the SOMMEPT cohort to assess whether TRNs display distinct autonomic dynamics before and after awakening. TRN awakenings were self‐marked with the wristband button and validated through electroencephalography (EEG) inspection by a psychiatrist and a sleep physician. Each TRN event (*N* = 412) was matched 1:1 to three control conditions: spontaneous awakenings in 60 healthy military participants, awakenings in 34 military PTSD patients without TRNs, and non‐nightmare awakenings from 74 military PTSD patients with TRNs. Pre‐awakening autonomic activity was analyzed over 10 min, and post‐awakening reactivity over a 2‐min window using nonparametric statistics with cluster‐based permutation correction.

**Results:**

TRNs were associated with heightened sudomotor activity and reduced vagal heart rate variability compared with spontaneous awakenings in healthy controls; differences were weaker versus PTSD patients without TRNs or non‐TRN awakenings. Phasic Electrodermal activity (EDA) showed earlier peaks and prolonged recovery, while movement was lower before TRN‐related awakenings. Post‐awakening, TRNs elicited an abrupt surge: heart rate accelerated within ~40 s and normalized by ~2 min; tonic EDA remained elevated, phasic EDA bursts were longer, slower to recover, and motor activity rose during the first ~80 s.

**Conclusions:**

TRNs display a distinctive autonomic pattern with pre‐awakening sudomotor buildup and post‐awakening cardiovascular–electrodermal surges, supporting biomarker‐based detection and targeted intervention.

**Trial registration:**

ClinicalTrials.gov Identifier: NCT04581850.

Trauma‐related nightmares (TRNs) are defined as vivid, recurring sleep phenomena linked to trauma, either through direct content or through emotionally charged mentation reflecting trauma‐associated affect.^1^, ^2^ They often recreate traumatic situations in striking detail, leading to abrupt and distressing awakening.^3^, ^4^, ^5^

TRNs are considered a core symptom of post‐traumatic stress disorder (PTSD), especially among military personnel.^6^ While the lifetime prevalence of nightmares in the general population is estimated at 3%–4%, rates increase in trauma‐exposed individuals^7^, ^8^, ^9^ and exceed 80% in military PTSD populations, with even higher prevalence in combat‐related.^7^, ^10^ Beyond frequency, TRNs disrupt sleep (fragmentation, hypervigilance, reduced efficiency) and promote maladaptive coping (e.g., alcohol/sedatives at bedtime).^6^, ^11^ Their presence worsens daytime PTSD symptoms, elevates the risk of depression and suicidality, and reinforces a vicious cycle of psychological and physiological dysregulation.^12^, ^13^ Current treatments only partially alleviate TRNs, and pharmacological options such as prazosin show mixed evidence across military populations,^14^, ^15^, ^16^ underscoring the need to clarify sustaining mechanisms to guide effective interventions.

However, objective physiological data remain scarce; most studies rely on self‐reports/diaries,^3^, ^13^ which are vulnerable to recall bias. Upon TRN‐related awakening, patients frequently report tachycardia, dyspnea, sweating, and a persistent sense of danger,^3^, ^4^ consistent with a sympathetic surge and the reactivation of trauma‐related interoceptive states.^2^, ^17^ Such experiences align with the broader physiology of PTSD, characterized by vagal dysregulation, low heart rate variability (HRV), and heightened sympathetic reactivity.^18^, ^19^

Yet, few studies have measured autonomic responses surrounding TRNs, with fewer conducted in ecologically valid conditions.^17^, ^20^, ^21^ Laboratory Polysomnography (PSG) documents sleep disruption in PTSD, but autonomic findings around TRNs remain inconsistent,^6^, ^22^ partly because lab settings may attenuate symptoms^23^, ^24^ and because similar autonomic responses can occur in nontraumatic awakenings.^25^

Nonetheless, advances in wearable sensor technologies allow high‐resolution, multi‐sensor monitoring of sleep and autonomic function in naturalistic environments. Wrist‐worn devices combined with portable Electroencephalography (EEG) can monitor Heart rate (HR), Electrodermal activity (EDA), temperature, and movement throughout the night. These data can be synchronized with subjective reports to capture the temporal and contextual dynamics of TRNs.^17^, ^20^, ^21^ Emerging evidence suggests that TRNs may provoke qualitatively distinct autonomic patterns—more intense, abrupt, and potentially involving greater electrodermal reactivity, particularly during Non‐rapid eye movement (NREM) sleep.^17^

To date, few studies have systematically compared TRN‐related awakenings to other types of awakenings within the same individual and in the same night, leaving a critical knowledge gap regarding the specificity of their physiological signature. The present study investigates whether TRNs elicit distinct autonomic dynamics before and after awakenings, compared to other nocturnal arousals in the same individual.

## Method

### Aim and Hypothesis

The primary aim of this study is to identify and characterize the autonomic signatures of TRNs in military service members with PTSD. It asks whether TRNs show distinct autonomic activation before and after awakenings compared to other nocturnal arousals.

#### Objective 1—Pre‐awakening dynamics

**Fig. 1:**
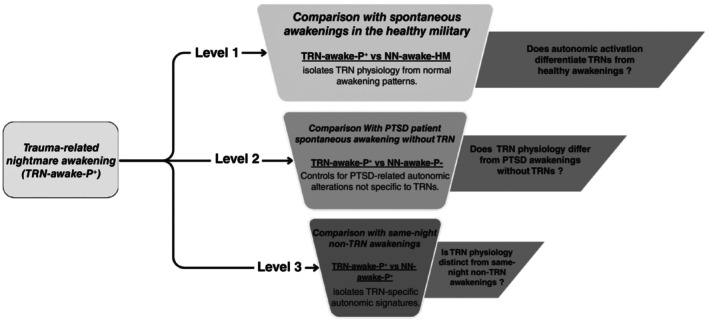
Funnel‐shaped analytical hierarchy comparing TRN‐related and non‐TRN awakenings. This figure illustrates the comparative analytical framework designed to progressively isolate the autonomic signatures of Trauma‐related nightmares (TRNs) awakenings. Each comparison level contrasts TRNs with a distinct category of matched non‐nightmare (NN) awakenings, allowing stepwise separation between nightmare‐specific physiology and patterns reflecting physiological awakening dynamics or general features of sleep in military patients with post‐traumatic stress disorder (PTSD). TRN‐awake‐P^+^, TRN‐related awakenings in military PTSD patients. NN‐awake‐HM, Non‐nightmare spontaneous awakenings in healthy military participants; NN‐awake‐P^−^, Non‐nightmare spontaneous awakenings in PTSD patients without any TRNs; NN‐awake‐P^+^, Non‐nightmare awakenings from the same nights as TRNs in PTSD patients (excluding events within ± 10 min of a TRN).

We assessed whether TRN‐related awakenings show greater pre‐awakening autonomic activation than matched non‐TRN awakenings. Comparisons followed a *funnel‐shaped hierarchy* (Fig. 1), reflecting the graded proximity of each awakening category to TRNs.

#### Objective 2—Post‐awakening reactivity

We assessed whether TRNs trigger a sharper autonomic response after awakening, compared to other non‐TRN awakenings within the same individuals.

### Participants

The PTSD (PTSD‐P) group comprised 130 veterans and active‐duty service members with combat‐related PTSD (PCL‐S > 44; no pretrauma psychiatric, sleep, or neurological disorders; sleep disturbances/TRNs not required for inclusion), recruited at five French military hospitals between November 2020 and March 2023.

Sixty‐five *healthy military* (HM) participants were screened *via* clinical interview and questionnaires to rule out PTSD and other disorders.

Ethical approval (EUDRACT 2020‐A01808‐31, RIPH2) was provided by the Ethics Committee (Comité de Protection des Personnes Sud‐Est 1) on August 28, 2020 (ref. 2020–80). All participants provided written informed consent.

### Study procedure, Clinical assessment, and Electrophysiological recordings

Participants underwent clinical evaluation before home‐based recordings. During overnight monitoring, they wore a Dreem 2 EEG headband and an Empatica E4 wristband. Data from both devices were synchronized, and protocol details are available in previous publications.^10^, ^17^

### Selection of TRN‐related awakenings

#### TRN‐awake‐P^+^−TRN‐related awakening of PTSD patients

TRN‐related awakenings required an awakening signaled by the participant using the E4 event button. At inclusion, participants were instructed to press the event button only after a TRN‐related awakening. Sleep diaries asked if a TRN occurred and if the button was pressed to cross‐verify each event.

Button‐marked events were synchronized across devices, cleaned (duplicates/consistency),^17^ and jointly reviewed by a psychiatrist and sleep physician to confirm EEG‐defined awakening and clinical coherence with TRNs. These events were classified as TRN‐awake‐P^+^.

For each validated TRN‐related awakening, the sleep stage preceding the event and the corresponding segment of the night (early, middle, or late) were extracted. These features were used for matching the non‐nightmare control awakenings.

### Selection of matched control awakenings

Non‐nightmare (NN) awakenings were defined as awakenings visible on EEG that were not signaled by a button press. In this PTSD cohort, where nightmares were considered trauma‐related, the absence of a button press indicated that the awakening was unlikely to be associated with a nightmare.

Matched Non‐nightmare awakenings (*NN*) were selected using a one‐to‐one procedure based on (i) the sleep stage awakening and (ii) the segment of the night (early, middle, late). A minimum duration of ≥30 s was imposed only for NN events to ensure stable signal quality, whereas TRN awakenings were clinically and EEG‐confirmed, making a temporal constraint unnecessary.

Within each pool of eligible NN events, the selection was random but constrained by the matching rules (sleep stage, night segment, and subject–night identity). Events already used for a previous match were excluded, and probabilistic weighting was applied to avoid overrepresentation of NN awakenings from participants or nights. This constrained‐random selection limits sampling bias while maintaining comparability of matched pairs.

#### NN‐awake‐HM–Non‐nightmare awakenings of HM participants

This group included spontaneous awakenings from the HM participants. For each TRN‐awake‐P^+^, one HM NN awakening (≥30 s) was selected 1:1 from >4500 events using constrained‐random sampling matched on pre‐awakening sleep stage and night segment, with exclusion of previously used events and probabilistic weighting to limit participant/night overrepresentation.

#### NN‐awake‐P^−^–Non‐nightmare awakenings of PTSD patients without TRNs

This group included NN awakenings from PTSD patients in whom no TRNs were objectively identified during the study (PTSD‐TRNs^−^). A similar 1:1 matching strategy was applied, using the same stage‐ and segment‐based matching constraints.

#### NN‐awake‐P^+^ − Non‐nightmare awakenings in PTSD patients with TRNs, drawn from the same nights as TRN events

To enable intra‐subject comparisons, NN awakenings were taken from the same nights as TRN‐awake‐P^+^ events. A ± 10‐min exclusion window prevented overlap between TRN and control awakenings. This choice was based on work,^21^ which examined autonomic dynamics during the 10 min preceding TRNs and has since been adopted in subsequent studies.^17^ Matching was restricted to sleep stage and night segment to ensure physiological comparability of awakenings, as these factors strongly shape autonomic dynamics.

Other confounders (e.g., age, sex, medication, alcohol) were accounted for at the group level, as including them in one‐to‐one matching would have reduced the sample size and statistical power. To conclude, four types of awakenings were investigated: NN‐awake‐HM (healthy military controls), NN‐awake‐P^−^ (PTSD without TRNs), NN‐awake‐P^+^ (same‐night non‐TRN awakenings), and TRN‐awake‐P^+^ (TRN‐related awakenings).

### Sleep and physiological parameters selection

Sleep architecture was extracted from Dreem EEG recordings and included standard macrostructural parameters (see Saguin *et al*.).^10^ Physiological data from the E4 wristband were used to assess autonomic dynamics. Parameters included:

- EDA: A custom analysis pipeline was applied to isolate tonic and phasic components following standard definitions of EDA.^26^, ^27^
  - Tonic skin conductance level (SCL) was derived using moving‐window smoothing (30‐s window) to extract trends, from which mean SCL, maximum SCL, and SCL area under the curve (AUC; μS · s) were computed.^26^
  - Phasic skin conductance responses (SCRs) were obtained by subtracting SCL from the EDA signal, and SCR peaks were detected as transient increases ≥0.01 μS with a minimum inter‐peak interval of 1 s, following the approach in Saguin et al.^17^ For each window time‐locked to awakening (*t* = 0), we extracted:
    - Number of SCR peaks; SCR peak latency (s): mean time from awakening (*t* = 0) to SCR peak; SCR response duration (s): interval between successive peaks or peak‐to‐window end; SCR recovery time (s): time from last SCR peak‐to‐window end; SCR area under the curve (AUC) (μS · s).
- Heart rate (HR) and heart rate variability (HRV) metrics included mean/max HR and standard indices: Standard deviation of NN interval (SDNN), RMSSD, pNN50 (time‐domain), LF and HF power (frequency‐domain). SDNN reflects overall variability; RMSSD and pNN50 index short‐term parasympathetic activity; HF power represents parasympathetic drive; and LF power reflects mixed sympathetic–parasympathetic modulation, providing a comprehensive autonomic profile when analyzed alongside EDA markers.
- Movement detection: Derived from the norm of triaxial accelerometry (*X*, *Y*, *Z*), capturing motor instability.

### Statistical analysis

#### General statistical procedures

Descriptive statistics included counts, percentages, means, and standard deviations. For sociodemographic and clinical variables, three groups (PTSD‐TRNs^+^, PTSD‐TRNs^−^, HM) were compared. Normality (Lilliefors test) and homogeneity of variances (Levene test) were assessed for each variable. When assumptions were met, a one‐way ANOVA was performed followed by Tukey–Kramer post hoc tests. Otherwise, Kruskal–Wallis tests were used, with Bonferroni‐corrected pairwise tests. Effect sizes were expressed as epsilon‐squared (ε^2^).

#### Pre‐awakening analysis

Pre‐awakening comparisons (Objective 1) followed a funnel‐shaped analytical structure (Fig. 1). This hierarchy contrasted TRN‐related awakenings (TRN‐awake‐P^+^) with three levels:

1. Level 1: spontaneous awakenings in healthy controls (NN‐awake‐HM),
2. Level 2: awakenings in PTSD patients without TRNs (NN‐awake‐P^−^), and
3. Level 3: non‐TRN awakenings occurring in the same individuals and on the same nights as TRNs (NN‐awake‐P^+^).

This design allows the isolation of TRN‐specific autonomic patterns from those reflecting normative awakening physiology or PTSD‐related sleep disruption.

A 10‐min pre‐awakening window was used, consistent with prior TRN work^17^, ^21^ and was chosen given the absence of established temporal standards for TRNs. As the onset and progression of physiological changes remain unknown, a 10‐min interval provides a conservative compromise—long enough to capture autonomic buildup while limiting the influence of unrelated fluctuations earlier in the night.

Aggregated physiological parameters were analyzed pairwise between each TRN‐related awakening and its matched non‐TRN counterpart. Paired *t*‐tests were used when the distribution of paired differences was normal, and Wilcoxon signed‐rank tests otherwise. As multiple physiological parameters were tested, *P*‐values were adjusted using Bonferroni correction across the family of parameters.

#### Post‐awakening analysis

For post‐awakening analyses (Objective 2), physiological values in the 2‐min window following each awakening were normalized (*z*‐score) using the 10‐min pre‐awakening baseline, enabling cross‐subject comparison of physiological reactivity. This 2‐min window, chosen due to the lack of established standards for post‐awakening reactivity duration, balances temporal resolution to capture rapid autonomic changes while minimizing artifacts from sleep–wake transitions or movement.

Time‐resolved group differences were then assessed using a cluster‐based permutation analysis (CPA). For each physiological variable, a two‐sample *t*‐statistic was computed at every 20‐s time bin comparing TRN‐related to non‐TRN awakenings. Time bins exceeding a predefined threshold (|*t*| >2) were grouped into clusters of consecutive bins. Following the approach of Maris & Oostenveld,^28^ each cluster was assigned a cluster statistic as the sum of its *t*‐values, capturing the strength and temporal extent of the effect.

To obtain the null distribution, group labels were randomly permuted 5000 times while preserving the temporal structure of the data. For each permutation, *t*‐values and cluster statistics were recomputed, and only the maximum cluster statistic (Tmax) was retained. The *P*‐value of each observed cluster was the proportion of permutations in which Tmax exceeded the observed cluster statistic, controlling the family‐wise error rate at the cluster level. Clusters were considered significant at *P* < 0.05 (two‐tailed).

All statistical analyses and visualizations were performed in MATLAB R2024a using custom scripts following best practices for time‐resolved data.

## Results

### Participants and awakenings selection

The study included SOMMEPT military participants: those with PTSD (PTSD‐P) and healthy controls (HM).

**Fig. 2:**
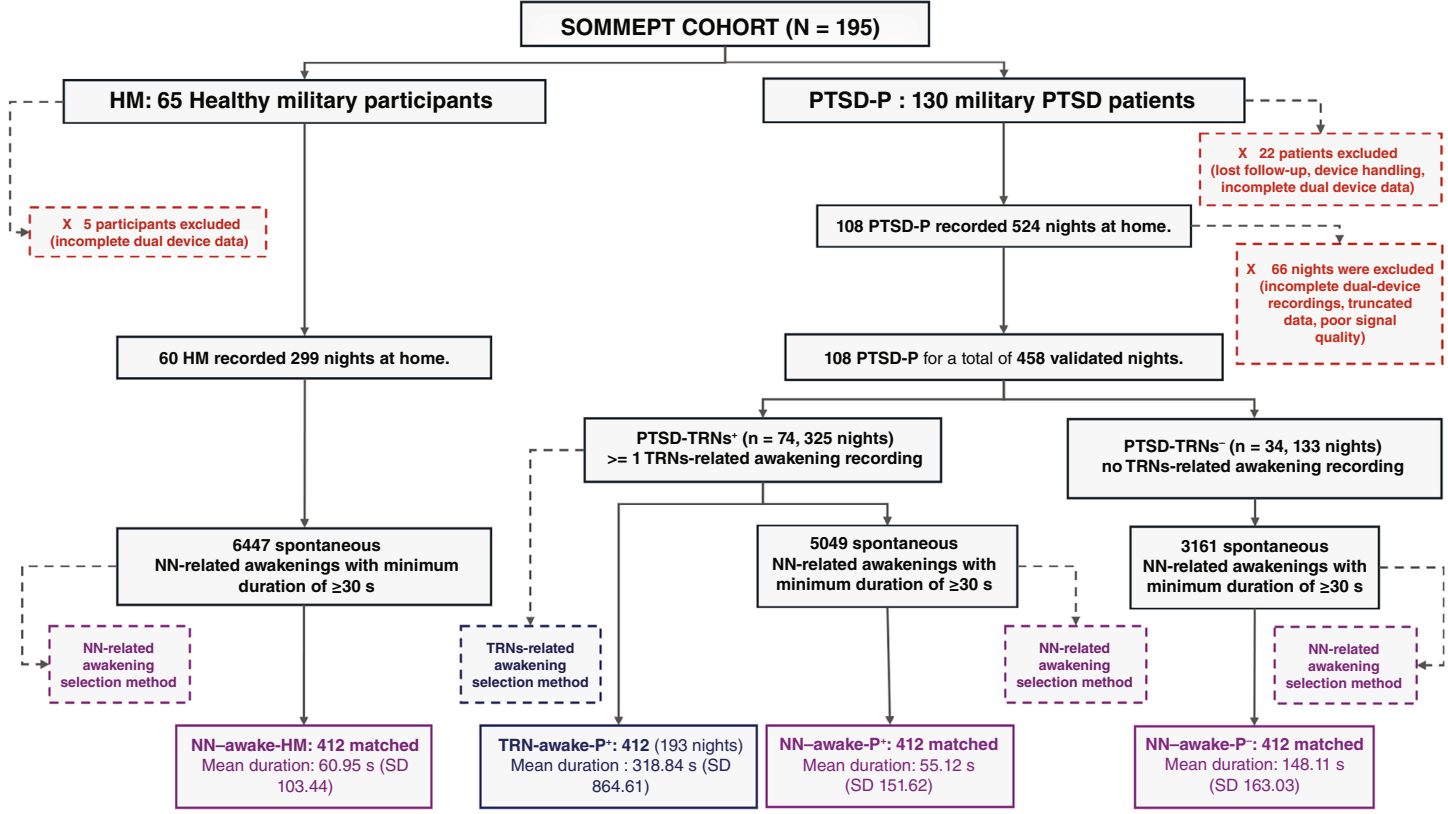
Flowchart of participant selection and awakening selection in the SOMMEPT cohort (*N* = 195). The figure shows the inclusion process, quality‐control exclusions, and final classification of Trauma‐related nightmares (TRNs) and Non‐nightmare (NN) awakenings. PTSD‐P: military PTSD patients; PTSD‐TRNs^+^, PTSD patients with ≥1 TRN‐related awakening; PTSD‐TRNs^−^, PTSD patients without TRNs awakenings objectively reported; HM: Healthy military group participants.

In PTSD‐P, 130 veterans were enrolled. After excluding 2 lost to follow‐up, 14 device issues, and 6 incomplete dual‐device datasets, 108 remained and contributed 524 nights. After quality control (48 incomplete dual‐device, 16 truncated, 2 poor quality), 458 nights were retained (Fig. 2). Overall, 74 patients (PTSD‐TRNs^+^, 68.5%) reported at least one TRN‐related awakening. They contributed 325 nights (mean 4.39/patient, ±1.44), with 412 TRN‐related awakenings (TRN‐awake‐P^+^) identified across 193 nights. This corresponds to 5.57 TRNs/patient (±5.98) and 1.27/night (8.89/week). Mean wake‐after‐nightmare (WAN) duration was 318.84 ± 864.61 s.

Across the same nights, 5049 awakenings ≥30 s were detected; 3781 were retained after filtering. 412 matched events (NN‐awake‐P^+^) were selected for intra‐subject comparison (mean duration 55.12 s, ±151.62).

The remaining 34 patients (PTSD‐TRNs^−^, 31.5%) contributed 133 nights (mean 3.91, ±1.27). Among 3161 awakenings ≥30 s, 412 matched events (NN‐awake‐P^−^) were selected (mean duration 148.11 s, ±163.03).

In HM, 60/65 (92.3%) completed dual‐device recordings, contributing 299 nights (mean 4.98 nights/participant, ±0.63). Overall, 6447 awakenings ≥30 s were identified, and 412 matched events (NN‐awake‐HM) were selected (mean duration 60.95 s, ±103.44).

### Sociodemographic and clinical characteristics of the selected participants

Sociodemographic and clinical characteristics of the overall sample (PTSD‐P and HM) have been previously reported.^10^ The following section presents the analytic sample:

#### Sociodemographic description

The PTSD‐TRNs^+^ group (*n* = 74; 70 men, 4 women; age 40.05 ± 8.40 years) and the PTSD‐TRNs^−^ group (*n* = 34; all men; age 42.03 ± 10.48 years) were compared to the HM group (*n* = 60; 54 men, 6 women; age 38.47 ± 9.01 years), with a significant age difference between PTSD‐TRNs^−^ and HM (*P* < 0.05, Bonferroni‐corrected).

Active‐duty status was 75.7% in PTSD‐TRNs^+^ and 70.6% in PTSD‐TRNs^−^ **versus** 100% in HM. Length of service was 18.0 ± 7.9 years (PTSD‐TRNs^+^), 18.6 ± 8.9 (PTSD‐TRNs^−^), and 14.2 ± 9.0 (HM). Sick leave at inclusion was 71.6% (PTSD‐TRNs^+^) and 61.8% (PTSD‐TRNs^−^) but absent in HM.

Operational history was similar: overseas deployments were 5.24 ± 4.74 (17.4 ± 17.6 months) in PTSD‐TRNs^+^, 5.26 ± 3.92 (19.2 ± 16.2) in PTSD‐TRNs^−^, and 4.55 ± 3.79 (17.0 ± 13.1) in HM. Traumatic events were 6.40 ± 11.02 (PTSD‐TRNs^+^), 9.40 ± 13.75 (PTSD‐TRNs^−^), versus 2.43 ± 4.53 (HM).

In PTSD‐TRNs^+^, exposure rates were highest for death (94.6%), injuries (62.2%), combat actions (51.4%), and explosions (45.9%), with lower frequencies for attacks (18.9%), assaults (16.2%), accidents (13.5%), and other trauma (8.1%).

PTSD‐TRNs^−^ showed a similar distribution, with death (88.2%), injuries (67.6%), combat (67.6%), and explosions (58.8%) predominating, and lower rates for attacks (23.5%), accidents (14.7%), assaults (14.7%), and other trauma (5.9%).

HM participants reported lower exposure, with explosions (35.0%), injuries (30.0%), and death (23.3%) most frequent, and combat (25.0%), assaults (16.7%), accidents (6.7%), or attacks (3.3%) less frequent; no participant reported other trauma.

#### Clinical description

PTSD symptom severity (PCL‐S: 47.01 ± 11.81 in PTSD‐TRNs^+^; 44.91 ± 15.33 in PTSD‐TRNs^−^ *vs*. 3.55 ± 8.44 in HM) and depressive symptoms (BDI: 13.89 ± 5.26; 14.32 ± 5.98 *vs*. 1.60 ± 2.31) were markedly higher in both PTSD groups.

Insomnia severity (ISI) and perceived sleep disturbances (PSQI) followed the same trend, with high scores in both PTSD‐TRNs^+^ (18.89 ± 3.25; 12.25 ± 4.07) and PTSD‐TRNs^−^ (17.13 ± 3.91; 11.82 ± 3.87), contrasting sharply with low scores in HM (3.47 ± 2.89; 3.55 ± 1.74). Symptoms suggestive of sleep‐related breathing disorders (Berlin questionnaire) were also elevated in both PTSD groups (1.68 ± 0.80 for PTSD‐TRNs^+^; 1.81 ± 0.83 for PTSD‐TRNs^−^), compared to lower scores in HM (0.57 ± 0.72).

No significant differences were observed between PTSD subgroups on clinical variables, except TRN frequency from TRNS‐FR,^29^ which was higher in PTSD‐TRNs^+^ (4.80 ± 5.18/week) than PTSD‐TRNs^−^ (3.10 ± 3.49/week; *P* < 0.05, Bonferroni‐corrected). Both significantly differed from the HM across all clinical measures.

Psychotropic medication use was common in both PTSD subgroups (absent in HM), with nearly all patients receiving ≥1 medication, mainly antidepressants and antipsychotics, and fewer receiving benzodiazepines, hypnotics, prazosin, or related agents. Only one drug differed between PTSD subgroups: alimemazine was more frequent in PTSD‐TRNs^−^ than PTSD‐TRNs^+^ (18.2% vs. 2.7%, *P*≈0.01). Full distributions are detailed in Table 1.

**Table 1:** Psychotropic medication profiles across PTSD subgroups

Medication category	PTSD‐TRNs^+^ (*n* = 74)	PTSD‐TRNs^−^ (*n* = 34)
Any psychiatric treatment	61 (83.6%)	29 (87.9%)
Antidepressant	50 (68.5%)	19 (57.6%)
Background neuroleptic	19 (26.0%)	7 (21.2%)
Short‐acting neuroleptic	23 (31.5%)	14 (42.4%)
Benzodiazepine	22 (30.1%)	4 (12.1%)
Hypnotic	16 (21.9%)	3 (9.1%)
Hydroxyzine	10 (13.7%)	5 (15.2%)
Alimemazine	2 (2.7%)*	6 (18.2%)*
Other anxiolytic	1 (1.4%)	0 (0.0%)
Mood stabilizer / antiepileptic	6 (8.2%)	2 (6.1%)
Prazosin	5 (6.8%)	1 (3.0%)

*Note*: Reports the distribution of prescribed psychotropic medication across PTSD patients with TRNs (PTSD‐TRNs^+^, *n* = 74), PTSD patients without TRNs (PTSD‐TRNs^−^, *n* = 34). Values are presented as *n* (%). Group comparisons were performed using pairwise Fisher exact tests. Statistically significant comparisons are marked with asterisks (*P < 0.05; **P < 0.01; ***P < 0.001).

### Sleep recordings profile across selected groups

Statistical differences were observed only between PTSD and HM. Reported *P*‐values reflect the omnibus Kruskal–Wallis test; unless stated otherwise, results remained significant after Bonferroni correction.

#### Sleep initiation and maintenance

Sleep onset latency (SOL) was significantly longer in both PTSD groups versus HM (29.77 ± 16.35 and 35.67 ± 32.07 vs. 17.46 ± 9.29 min; *P* < 0.001, *ε* ^2^ = 0.150). Wake after sleep onset (WASO) was higher in PTSD‐TRNs^+^ than HM (31.69 ± 21.02 vs. 22.26 ± 14.10 min; *P* < 0.01, *ε* ^2^ = 0.054), while the PTSD‐TRNs^−^ contrast was not significant. Sleep efficiency (SE) was reduced in both PTSD groups compared with HM (86.57 ± 5.30% and 84.08 ± 10.02% vs. 90.66 ± 4.05%; *P* < 0.001, *ε* ^2^ = 0.163). Total sleep time (TST) did not differ significantly across groups (409.83 ± 71.24; 374.17 ± 69.45; 399.77 ± 53.32 min).

#### Sleep architecture

N1 and N3 did not differ significantly across groups (N1: 7.29 ± 2.75%, 7.94 ± 4.40%, 6.82 ± 2.06%; — N3: 17.68 ± 7.71%, 17.43 ± 8.58%, 19.78 ± 7.11%). N2 was higher in PTSD‐TRNs^+^ than HM (50.85 ± 8.54% vs. 46.29 ± 6.69%; *P* < 0.05, *ε* ^2^ = 0.044), with PTSD‐TRNs^−^ showing intermediate, nonsignificant values (50.42 ± 10.58%).

REM sleep was lower in PTSD‐TRNs^+^ than HM (24.19 ± 6.77% vs. 27.11 ± 5.03%; *P* < 0.05, ε^2^ = 0.043), while PTSD‐TRNs^−^ did not differ significantly (24.20 ± 7.71%).

NREM proportions were higher in PTSD‐TRNs^+^ than HM (75.81 ± 6.77% vs. 72.89 ± 5.03%; *P* < 0.05, *ε* ^2^ = 0.043), while PTSD‐TRNs^−^ did not differ significantly (75.80 ± 7.11%).

Fragmentation indices were comparable across all groups, with global fragmentation averaging 6.13 ± 2.52 in PTSD‐TRNs^+^, 6.67 ± 2.93 in PTSD‐TRNs^−^, and 5.94 ± 1.59 in HM. Similar patterns were observed for REM fragmentation (9.81 ± 4.15, 9.45 ± 3.06, and 9.42 ± 2.74, respectively) and for NREM fragmentation (6.11 ± 2.62, 6.82 ± 3.01, and 5.78 ± 1.51, respectively), with no significant group differences.

### Physiological 10 min pre‐awakening dynamics across groups: intergroup comparison of TRNs‐related and non‐TRN‐related awakenings

As illustrated in Fig. 1, intergroup comparisons followed the funnel‐shaped framework (Levels 1–3). This approach was designed to gradually isolate TRN‐specific physiological dynamics from general PTSD‐related or normative awakening physiology.

#### Level 1: TRN‐awake‐P^+^ vs. NN‐awake‐HM

We compared TRN‐related awakenings (TRN‐awake‐P^+^, *N* = 412) to spontaneous awakenings in healthy controls (NN‐awake‐HM, *N* = 412) using 10‐min pre‐awakening data. Reported effects remained significant after Bonferroni correction unless stated otherwise.

TRN‐awake‐P^+^ showed higher autonomic arousal than NN‐awake‐HM: mean HR was higher, while HRV indices (SDNN, RMSSD, pNN50) and LF power were lower. Tonic EDA differed most, with higher mean, maximum, and AUC SCL. Phasic EDA also differed (shorter SCR peak latency, longer recovery), while pre‐awakening movement was lower.

#### Level 2: TRN‐awake‐P^+^ vs. NN‐awake‐P^−^

To test whether these patterns were TRN‐specific rather than reflecting PTSD‐related sleep, we compared TRN‐awake‐P^+^ to awakenings in PTSD patients without TRNs (NN‐awake‐P^−^, *N* = 412).

Between these two PTSD subgroups, pre‐awakening tonic EDA remained discriminant: max SCL was significantly higher before TRNs, whereas other SCL indices showed only trends. HR metrics did not differ, but HRV provided more contrast. TRN‐awake events were associated with higher pNN50 and increased LF power, reflecting distinct short‐term vagal dynamics. Other HRV indices showed only nominal or non‐significant differences. Movement and SCR temporal features were slightly altered in TRN‐awake, but these effects did not survive correction. Full statistics for level 1 & 2 comparisons are provided in Table 2, with additional graphical views available in Supplementary materials S1 and S2.

**Table 2:** Physiological comparison of TRN‐related awakenings to two control conditions (level 1 & 2)

Parameters	Level 1 NN‐awake‐HM (*N* = 412)	Level 2 NN‐awake‐P^−^ (*N* = 412)	TRN‐awake‐P^+^ (*N* = 412)	*P*‐value (TRN‐awake‐P^+^ vs. NN‐awake‐HM)	*P*‐value (TRN‐awake‐P^+^ vs. NN‐awake‐P^−^)
Heart rate (HR)					
Mean HR (bpm)	**64.36 (±13.72)**	69.07 (±17.41)	**67.91 (±17.48)**	**< 0.01 (ε** ^**2**^ **= 0.023)** *	0.073 (ε^2^ = 0.008)
Max HR (bpm)	76.46 (±22.05)	78.98 (±24.15)	78.47 (±24.57)	0.212 (ε^2^ = 0.004)	0.183 (ε^2^ = 0.004)
Heart rate variability (HRV)					
Number of beats	365.59 (±254.87)	**399.22 (±275.31)**	**348.31 (±269.44)**	0.339 (ε^2^ = 0.002)	**<0.01 (ε** ^**2**^ **= 0.022)** *
SDNN (s)	**0.062 (±0.051)**	0.044 (±0.033)	**0.048 (±0.0420)**	**<0.001 (ε** ^**2**^ **= 0.035)** *	0.205 (ε^2^ = 0.004)
RMSSD (s)	**0.053 (±0.044)**	**0.035 (±0.029)**	**0.040 (±0.037)**	**<0.001 (ε** ^**2**^ **= 0.045)** *	**<0.05 (ε** ^**2**^ **= 0.012)**
*P*‐NN50 (%)	**23.09 (±21.37)**	**11.52 (±14.49)**	**16.70 (±18.82)**	**<0.001 (ε** ^**2**^ **= 0.045)** *	**<0.001 (ε** ^**2**^ **= 0.045)** *
LF (s^2^)	**0.0018 (±0.030)**	**0.00047 (±0.0073)**	**0.016 (±0.202)**	**<0.001 (ε** ^**2**^ **= 0.069)** *	**<0.001 (ε** ^**2**^ **= 0.032)** *
HF (s^2^)	**0.00017 (±0.0029)**	2.63e‐05 (±0.00023)	**0.00051 (±0.0058)**	**<0.05 (ε** ^**2**^ **= 0.010)**	0.0657 (ε^2^ = 0.008)
Electrodermal activity (EDA)					
Skin conductance level (EDA SCL)					
Mean EDA SCL (μS)	**1.39 (±2.64)**	**2.41 (±5.73)**	**3.87 (±8.72)**	**<0.001 (ε** ^**2**^ **= 0.067)** *	**<0.01 (ε** ^**2**^ **= 0.020)**
Max EDA SCL (μS)	**1.80 (±3.24)**	**2.89 (±6.38)**	**4.62 (±9.55)**	**<0.001 (ε** ^**2**^ **= 0.059)** *	**<0.01 (ε** ^**2**^ **= 0.023)** *
Area under the curve EDA SCL (μS·s)	**213.26 (±494.69)**	**261.74 (±613.89)**	**385.60 (±787.12)**	**< 0.001 (ε** ^**2**^ **= 0.032)** *	**< 0.01 (ε** ^**2**^ **= 0.017)**
Skin conductance response (EDA SCR)					
Number of EDA SCR peaks	21.81 (±25.785)	24.32 (±29.83)	25.29 (±29.92)	0.372 (ε^2^ = 0.002)	0.606 (ε^2^ = 6.43e‐04)
Peak latency (s)	**306.61 (±158.98)**	**289.47 (±154.40)**	**267.92 (±146.41)**	**<0.001 (ε** ^**2**^ **= 0.031)** *	**< 0.05 (ε** ^**2**^ **= 0.010)**
Response duration (s)	63.41 (±119.274)	82.48 (±159.00)	77.21 (±146.85)	0.426 (ε^2^ = 0.002)	0.583 (ε^2^ = 7.31e‐04)
Recovery time (s)	**49.11 (±99.81)**	72.49 (±119.28)	**81.99 (±136.68)**	**<0.001 (ε** ^**2**^ **= 0.027)** *	0.765 (ε^2^ = 2.17e‐04)
Area under the curve (EDA SCR) (μS·s)	0.133 (±0.182)	0.123 (±0.175)	0.137 (±0.175)	0.744 (ε^2^ = 2.58e‐04)	0.152 (ε^2^ = 0.005)
Movement					
Total movement (a.u.)	**9.14 (±5.177)**	**8.74 (±5.097)**	**8.19 (±6.304)**	**<0.001 (ε** ^**2**^ **= 0.035)** *	**<0.01 (ε** ^**2**^ **= 0.017)** *

*Note*: Reports Levels 1 and 2 of the funnel‐shaped analysis. Mean values (± standard deviation) of physiological parameters extracted from the 10 min preceding TRN‐related awakenings in PTSD patients (TRN‐awake‐P^+^, *N* = 412) are compared to (1) spontaneous awakenings in healthy military participants (NN‐awake‐HM; *N* = 412), and (2) awakenings in PTSD patients who did not report any TRNs across all recorded nights (NN‐awake‐P^−^; *N* = 412). Comparisons were performed using Kruskal–Wallis tests, with associated effect sizes (*ε* ^2^) reported. Statistically significant *P*‐values (<0.05) are indicated.

* Denotes results that remained significant after Bonferroni correction.

#### Level 3: TRN‐awake‐P^+^ *vs.* NN‐awake‐P^+^

We contrasted TRN‐awake‐P^+^ (*N* = 412) with same‐night non‐nightmare awakenings from the same patients (NN‐awake‐P^+^, *N* = 412) over the 10‐min pre‐awakening window.

Mean HR was slightly lower in TRN‐awake (67.92 ± 17.49 bpm) than in NN‐awake‐P^+^ (68.75 ± 16.02 bpm, *P* < 0.01, *ε* ^2^ = 0.023). HRV showed higher vagal indices: SDNN (0.0488 ± 0.0421 s vs. 0.0445 ± 0.0432 s; *P* < 0.05), RMSSD (0.0409 ± 0.0380 s vs. 0.0379 ± 0.0396 s; *P* < 0.05), and pNN50 (16.70 ± 18.83% vs. 14.95 ± 18.83%; *P* < 0.05) (*ε* ^2^≈0.012–0.014). Spectral HRV showed higher LF (0.0165 ± 0.2027 s^2^ vs. 0.0021 ± 0.0253 s^2^; *P* < 0.05, *ε* ^2^ = 0.014) and HF power (0.00051 ± 0.00581 s^2^ vs. 0.00003 ± 0.00016 s^2^; *P* < 0.05, *ε* ^2^ = 0.010).

Tonic EDA was higher in TRN‐awake, both in mean SCL (3.87 ± 8.73 μS vs. 2.68 ± 4.72 μS, *P* < 0.01, *ε* ^2^ = 0.019) and max SCL (4.62 ± 9.55 μS vs. 3.46 ± 5.94 μS, *P* < 0.05, *ε* ^2^ = 0.014). EDA SCR peak latency was shorter before TRNs (267.92 ± 146.41 s vs. 296.59 ± 154.51 s, *P* < 0.05, *ε* ^2^ = 0.012); response duration (77.22 ± 146.85 s vs. 63.09 ± 129.98 s, *P* < 0.05, *ε* ^*2*^ = 0.014) and recovery time (81.99 ± 136.69 s vs. 59.58 ± 111.59 s, *P* = 0.096, *ε* ^2^ = 0.007) were slightly longer.

Finally, pre‐awakening movement was lower in TRN‐awake (8.19 ± 6.30 a.u.) vs. NN‐awake‐P^+^ (9.89 ± 9.27 a.u.; *P* < 0.01, *ε* ^2^ = 0.020).

**Fig. 3:**
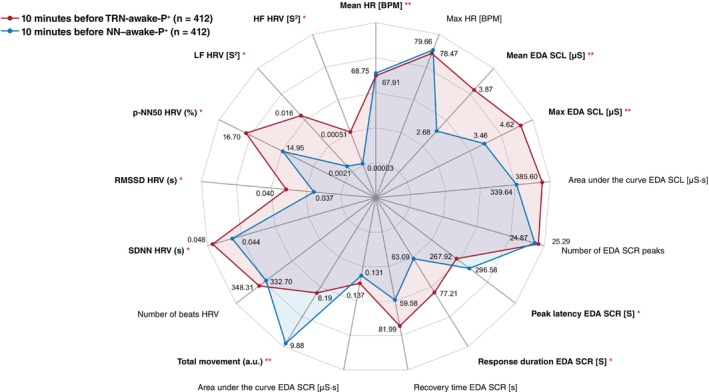
Physiological signature preceding TRN‐awake‐P^+^ vs. NN‐awake‐P^+^ awakenings Comparison of physiological profiles during the 10 min preceding TRN‐related awakenings in PTSD patients (TRN‐awake‐P^+^, *N* = 412, in red) and non‐nightmare awakenings from the same nights (NN‐awake‐P^+^, *N* = 412, in blue). Each axis of the spider plot represents a physiological variable measured in the 10 min before awakening: Heart rate (HR), Heart rate variability (HRV), tonic and phasic electrodermal activity (EDA, SCL, and SCR), and body movement. HR reflects cardiac arousal; HRV indices capture parasympathetic and sympathetic balance; tonic and phasic EDA index sympathetic activation; and movement reflects motor activity surrounding awakening. Red asterisks indicate statistically significant differences between conditions (*P* < 0.05, <0.01, <0.001), computed using Kruskal–Wallis tests with Bonferroni correction for multiple comparisons. Values plotted correspond to mean values per group.

However, none of these differences remained significant after Bonferroni correction. Other variables—including max HR, SCR peak count, AUC SCL/SCR, and total beats—did not differ. Full results are presented in Fig. 3.

### Awakening‐related physiological activation: comparing TRNs and non‐TRNs events within‐night and within‐subject

**Fig. 4:**
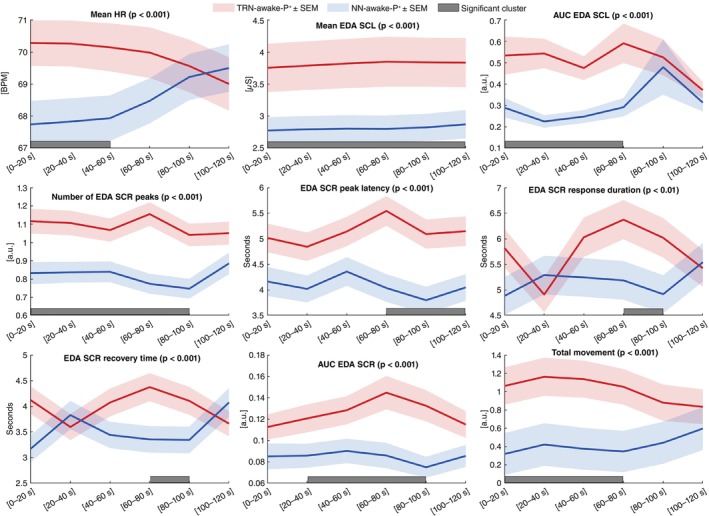
Temporal dynamics of acute physiological responses to TRN‐awake‐P^+^ vs. NN‐awake‐P^+^. Time‐resolved analysis of nine normalized physiological variables following awakenings, comparing TRN‐related awakenings (TRN‐awake‐P^+^, red) with non‐TRNs awakenings occurring during the same nights and in the same patients (NN‐awake‐P^+^, blue). For each variable, mean values and standard errors (±SEM) are plotted over the 2‐min post‐awakening period, binned in 20‐s epochs. Gray bars indicate significant time clusters identified using a nonparametric cluster‐based permutation test (*n* = 5000 permutations, cluster threshold *t* > 2, two‐tailed). Reported *P*‐values correspond to the empirical significance level of the largest cluster for each variable. This approach controls for multiple comparisons across time while preserving the temporal structure of physiological reactivity. Variables include Heart rate (HR), Electrodermal activity (EDA SCL (Tonic) and EDA SCR (Phasic) parameters), and Movement.

Post‐awakening dynamics were compared between TRN‐awake‐P^+^ (*N* = 412) and NN‐awake‐P^+^ (*N* = 412) using within‐subject cluster‐based permutation testing over the 2‐min after awakening (Fig. 4).

For HR, TRN‐awake‐P^+^ showed a significant acceleration during the first minute post‐awakening, peaking within 40 s and returning by 2 min. Mean HR was higher than NN‐awake‐P^+^ during the first 60 s (70.23 vs. 67.84 bpm; *Δ* = +2.40 bpm, 95% CI [1.24, 3.56], *d* = 0.16, *P* < 0.001).

EDA tonic responses (SCL) were elevated across the 2‐min window. Mean SCL was consistently higher in TRN‐awake‐P^+^ (3.82 vs. 2.81 μS; *Δ* = +1.00 μS, 95% CI [0.65, 1.35], *d* = 0.16, *P* < 0.001), with greater cumulative activity from 0–80 s (0.54 vs. 0.26; *Δ* = +0.27, 95% CI [0.19, 0.36], *d* = 0.22, *P* < 0.001).

For phasic EDA (SCR), TRN‐awake‐P^+^ showed more frequent and prolonged responses. The mean number of peaks was higher over 0–100 s (1.10 vs. 0.81; Δ = +0.29, 95% CI [0.22, 0.37], *d* = 0.24, *P* < 0.001). Dynamics were slower: peak latency was longer (5.26 *vs*. 3.96 s; *Δ* = +1.30 s, 95% CI [0.86, 1.74], *d* = 0.23, *P* < 0.001), response duration was greater (6.20 vs. 5.05 s; *Δ* = +1.14 s, 95% CI [0.40, 1.89], *d* = 0.15, *P* < 0.01), and recovery time was slower (4.24 vs. 3.35 s; Δ = +0.89 s, 95% CI [0.37, 1.41], *d* = 0.17, *p* < 0.01). SCR AUC was larger from 20–100 μS·s (0.132 vs. 0.084; Δ = +0.047, 95% CI [0.030, 0.065], *d* = 0.18, *P* < 0.001).

Finally, body movements were more pronounced following TRN‐awake‐P^+^ during the first 80 s (1.10 vs. 0.37 a.u.; Δ = +0.74, 95% CI [0.44, 1.04], *d* = 0.17, *p* < 0.001), consistent with heightened motor activation.

## Discussion

This study aimed to identify autonomic signatures of trauma‐related nightmares (TRNs) in military PTSD and whether they differ from other awakenings. Using home‐based sensor recordings and a hierarchical comparison design, we examined pre‐ and post‐awakening autonomic dynamics in a large, well‐characterized cohort.

More than 750 nights of dual‐device recordings were analyzed from PTSD patients and HM controls. In PTSD, 74 patients experienced at least one TRN, yielding 412 objectively marked TRN‐related awakenings—among the largest TRN datasets to date.^17^, ^20^, ^21^

Tonic EDA consistently differentiated TRN‐related from non‐TRN awakenings across all levels, indicating a stable pre‐awakening signature. HRV differences were less consistent, appearing clearly when contrasting TRNs with awakenings from individuals without TRNs and diminishing in the within‐subject comparison. Pre‐awakening HR and movement showed limited divergence, with their strongest differentiation emerging after awakening rather than before. Together, these findings delineate a stepped pattern across the funnel hierarchy: EDA was the most robust pre‐awakening marker of TRNs, HRV reflected a more graded PTSD‐related vulnerability, and HR and movement primarily captured the acute response at awakening rather than anticipatory processes.

### Sleep macrostructure: similar profiles in PTSD subgroups despite TRNs

An important and unexpected finding is the absence of macrostructural sleep differences between PTSD patients with (PTSD‐TRNs^+^) and without (PTSD‐TRNs^−^) recorded TRNs. Despite the presence of TRNs, sleep initiation, efficiency, architecture, and fragmentation were comparable, suggesting that TRN occurrence is not reflected at the macrostructural level but rather relates to broader PTSD‐associated processes such as hyperarousal, comorbid insomnia, or sleep‐disordered breathing.^6^, ^20^, ^30^

### Influence of psychotropic medication

Psychotropic medications—such as SSRIs, benzodiazepines, antipsychotics, and adrenergic receptor antagonists like prazosin—modify autonomic function in PTSD.^15^ Their use was common across both PTSD subgroups, with similar prescription patterns and no systematic differences in major drug classes that might influence cardiovascular or electrodermal regulation. The only significant contrast concerned alimemazine (more common in PTSD‐TRNs^−^), while prazosin, benzodiazepines, hypnotics, and other sedatives were comparable across PTSD groups. This makes it unlikely that autonomic differences between PTSD‐TRNs^+^ and PTSD‐TRNs^−^, or between TRN‐related and NN awakenings, reflect differential exposure to these drugs. Nonetheless, treatments may have attenuated or reshaped response amplitudes; future studies stratifying by class and dose are needed to quantify their influence on nocturnal autonomic dynamics in TRNs.

### Pre‐awakening dynamics: sustained EDA activation and vagal withdrawal

In the minutes preceding TRN‐related awakenings, tonic EDA was elevated and vagally mediated HRV reduced, indicating a shift toward sympathetic dominance and supporting EDA as the most sensitive anticipatory marker.

This profile aligns with interoceptive dysregulation models of PTSD,^2^ which propose that a buildup of bodily threat states during sleep may contribute to nightmare generation. Reduced vagal tone may reflect impaired prefrontal inhibition of limbic–brainstem circuits, while elevated sudomotor drive indicates sympathetic arousal.^6^, ^31^, ^32^, ^33^, ^34^ These results extend previous findings of pre‐awakening EDA peaks,^17^ and fit broader evidence of autonomic hyperresponsivity in trauma‐exposed populations.^35^, ^36^, ^37^, ^38^, ^39^ In military contexts, where hypervigilance is reinforced by operational constraints, this anticipatory arousal may be particularly pronounced.^11^

### Post‐awakening dynamics: abrupt multisystem activation

In contrast to gradual pre‐event buildup, the post‐awakening period showed an abrupt surge: HR rose within 40 s and normalized by 2 min, phasic EDA increased with longer recovery, and motor activity rose during the first 80 s.

This two‐phase pattern—pre‐awakening sudomotor buildup followed by post‐awakening cardiovascular and motor activation—suggests sequential recruitment of autonomic subsystems. Pre‐event buildup may facilitate TRNs onset, while post‐event surges reflect defensive mobilization. Repeated across nights, this loop may sustain hypervigilance and maladaptive sleep behaviors, contributing to fragmented sleep, impaired mood, and reinforced hyperarousal in chronic PTSD.^6^, ^11^

### Neurophysiological interpretation

A strength of this study is its funnel‐shaped design, which isolated TRN‐specific alterations from broader influence. By contrasting TRNs with awakenings from healthy controls, PTSD patients without TRNs, and same‐night awakenings from the same patients, we showed that electrodermal and vagal alterations persisted under stringent control. This supports the potential specificity of the physiological profile observed in TRNs rather than general vulnerability to PTSD.^17^, ^20^, ^21^, ^24^

Within this framework, sustained electrodermal activation and reduced vagally mediated cardiovascular variability may reflect persistent sympathetic tone and reduced vagal flexibility, in line with neurovisceral integration models linking prefrontal regulation, emotional control, and cardiac variability.^31^, ^32^, ^40^ Converging HRV indices (despite limitations of LF powe^41^, ^42^), indicate a shift toward sympathetic dominance before TRN awakenings. Following arousal, cardiovascular acceleration and motor release emerge abruptly, consistent with locus coeruleus–amygdala discharges mediating rapid defensive mobilization.^33^

The dissociation between sudomotor pre‐activation and cardiovascular post‐activation may reflect distinct but interacting mechanisms: insula‐driven interoceptive prediction errors may amplify electrodermal output before awakening,^43^, ^44^ while baroreflex buffering constrains cardiac responses until noradrenergic discharges from the locus coeruleus trigger mobilization after awakening. This temporal sequence may reflect a transition from an unconscious bodily state to a conscious emotional reaction at awakening, consistent with neurocognitive models of nightmare distress in which emotional overload emerges at awakening.^3^, ^6^, ^45^

EDA, linked to sympathetic sudomotor output, could index a preconscious corporeal load,^26^, ^46^ whereas cardiovascular alterations may capture the shift to conscious fear and the realization of TRN mentation.^31^, ^47^ Together, these findings align with prior descriptions of autonomic buildup before TRNs,^48^ and with evidence of sleep‐related motor suppression interpreted as a freezing‐like response in trauma‐exposed populations.^49^ By contrast, reduced pre‐awakening motor activity may appear at odds with reports of large‐amplitude motor behaviors and REM sleep without atonia (RWA) described in PSG studies of military PTSD patients.^50^

These findings can be reconciled by several factors: RWA‐associated motor episodes are primarily described during REM sleep, whereas most TRNs in chronic PTSD occur during NREM sleep,^17^ where motor atonia is preserved and overt enactment is less likely; dramatic motor behaviors reported retrospectively^3^ may not reflect continuous pre‐awakening motor discharge, as freezing‐like suppression can precede conscious awareness; and subtle atonia loss or focal twitches detectable by PSG EMG may remain undetected by wrist‐worn accelerometry.

Importantly, the absence of pre‐awakening motor activation does not contradict REM‐focused observations but reflects sleep‐stage distribution, methodological sensitivity, and the distinction between subjective motor experiences and objectively measurable movement in naturalistic settings. These nuances do not alter the core temporal pattern observed, which aligns with trauma memory network models in which sensory and bodily fragments of traumatic experience are reactivated during sleep and overwhelm regulatory systems at awakening.^20^, ^51^

Taken together, these findings suggest that in TRNs, pre‐awakening activity is primarily expressed through sudomotor channels, while motor and cardiovascular responses remain inhibited until awakening, when autonomic discharges may amplify an ongoing TRN or trigger the experience.

### Clinical and translational implications

First, the pre‐awakening phase offers a window for prediction and preventive intervention. Algorithms combining sustained SCL elevation and reduced HRV could be embedded in wearables to trigger alerts or adaptive biofeedback.^17^ Second, the post‐awakening surge highlights the need for strategies to accelerate autonomic downregulation—paced breathing, vagal stimulation, or sensory grounding—to facilitate sleep resumption and limit sympathetic inertia. Integration with existing evidence‐based therapies, such as imagery rehearsal therapy or prazosin, could help identify patients who may benefit most from combined physiological and cognitive‐behavioral approaches.^52^ Third, the reproducibility of TRN‐specific markers across three levels of comparison supports their potential as biomarkers for diagnosis and monitoring, with possible extension to other disorders featuring pathological nocturnal arousals.

Finally, TRNs may provide a transdiagnostic model of nocturnal autonomic dysregulation, combining a reproducible anticipatory phase with a distinct post‐awakening arousal response. Applying this temporal framework to idiopathic nightmare disorder and other disorders of abnormal arousal (e.g., nocturnal panic, confusional arousals) may help refine nosological distinctions and inform individualized interventions.

### Limitations

Several limitations should be acknowledged. The absence of PSG precluded assessing respiratory and muscle activity, which may contribute to autonomic activation. This limitation is particularly relevant given the associations between TRNs, sleep‐disordered breathing, and nocturnal autonomic arousal in PTSD. While the present home‐based, multi‐night protocol prioritized feasibility and ecological validity, future studies incorporating complementary measures of nocturnal respiration could clarify respiratory contributions to TRN‐related autonomic dynamics.

Beyond respiratory factors, the study focused on autonomic physiology and did not include pre‐awakening EEG contrasts. Although home EEG was collected and reviewed to validate each awakening, and prior work has suggested EEG markers of sleep “lightening” before nightmare‐related awakenings,^17^, ^20^, ^21^, ^30^ the present approach prioritized autonomic signals for scalability and clinical translation. Integrating EEG dynamics in future work may nonetheless provide complementary insights and refine the temporal characterization of TRNs.

At the methodological level, TRN identification relied on manual button presses which, despite dual clinical validation, may have introduced temporal imprecision. This constraint, combined with the choice of 10‐min pre‐awakening and 2‐min post‐awakening windows, reflects a compromise in the absence of consensus regarding the temporal boundaries of TRN‐related physiological changes. Determining optimal temporal windows remains an open challenge. In addition, although awakenings without a button press were considered unlikely to reflect nightmare experiences in this PTSD cohort, occasional missed reports cannot be excluded (e.g., omission, low awareness, or rapid return to sleep), such that a small number of TRN‐related events may have been included among the control awakenings.

Finally, the use of wearable‐based recordings in naturalistic settings implies sensitivity to body movement, which can influence autonomic signals such as HR and EDA. Although movement was lower—not higher—prior to TRNs, making it unlikely to account for the observed autonomic differences, motion‐related artifacts cannot be excluded. Because motor agitation is part of the behavioral expression of TRNs, strong correction for movement would risk removing meaningful variance; future studies using multi‐sensor fusion approaches may better dissociate physiological activation from motion‐induced artifacts.

## Conclusions

This study characterizes a temporally structured physiological signature of TRNs in military personnel with PTSD. These events combine sustained pre‐awakening autonomic activation with an abrupt post‐awakening surge. The persistence of these differences across three comparison levels—including within‐night, within‐subject contrasts—supports their specificity to TRNs rather than general PTSD vulnerability. By integrating temporal dynamics with hierarchical comparisons, these findings support the development of real‐time detection and targeted interventions and suggest that TRNs may represent a useful model of autonomic dysregulation. This approach bridges physiological mechanisms and clinical translation, with potential implications for biomarker‐based, personalized care in trauma‐related sleep disturbances.

## Author contributions

Dorone Feingold: Conceptualization, Data analysis, Interpretation, Software. Michael Quiquempoix: Conceptualization, Data analysis supervision. Marion Remadi: Sleep scoring. Gilles Sipahimalani: Medical recruitment. Bertrand Lahutte: Conceptualization. Damien Léger: Conceptualization Danielle Gomez‐Merino: Conceptualization. Mounir Chennaoui: Conceptualization, Supervision. Emeric Saguin: Conceptualization, Interpretation, Supervision.

## Funding

This research was supported by the French Armed Forces Health Service (Grant no. 2020PRI01).

## Disclosure statement

The authors declare no conflict of interest.

## Supporting information


**Figure S1.** Physiological signature preceding TRN‐awake‐P^+^ versus NN‐awake‐HM awakenings. Comparison of physiological profiles during the 10 min preceding trauma‐related nightmare (TRN)‐related awakenings in PTSD patients (TRN‐awake‐P^+^, *N* = 412, in red) and spontaneous awakenings in healthy military controls (NN‐awake‐HM, *N* = 412, in blue). Each axis of the spider plot represents a physiological variable measured in the 10 min before awakening: Heart rate (HR), Heart rate variability (HRV), tonic and phasic Electrodermal activity (EDA, SCL, and SCR), and body movement. HR reflects cardiac arousal; HRV indices capture parasympathetic and sympathetic balance; tonic and phasic EDA index sympathetic activation; and movement reflects motor activity surrounding awakening. Red asterisks indicate statistically significant differences between conditions (*P* <0.05, <0.01, <0.001), computed using Kruskal–Wallis tests with Bonferroni correction for multiple comparisons. Values plotted correspond to mean values per group.

**Figure S2.** Physiological signature preceding TRN‐awake‐P^+^ versus NN‐awake‐P^−^ awakenings. Comparison of physiological profiles during the 10 min preceding trauma‐related nightmare (TRN)‐related awakenings in PTSD patients (TRN‐awake‐P^+^, *N* = 412, in red) and non‐nightmare awakenings in PTSD patients without any TRN awakenings (NN‐awake‐P^−^, *N* = 412, in blue). Each axis of the spider plot represents a physiological variable measured in the 10 min before awakening: Heart rate (HR), Heart rate variability (HRV), tonic and phasic Electrodermal activity (EDA, SCL, and SCR), and body movement. HR reflects cardiac arousal; HRV indices capture parasympathetic and sympathetic balance; tonic and phasic EDA index sympathetic activation; and movement reflects motor activity surrounding awakening. Red asterisks indicate statistically significant differences between conditions (*P* <0.05, <0.01, <0.001), computed using Kruskal–Wallis tests with Bonferroni correction for multiple comparisons. Values plotted correspond to mean values per group.

## Data Availability

The data supporting the findings of this study are available from the corresponding author upon reasonable request.

## References

[1] Davis JL . Treating Post‐Trauma Nightmares: A Cognitive Behavioral Approach. Springer Publishing Company, New York, NY, 2008.

[2] Levin R , Nielsen TA . Disturbed dreaming, posttraumatic stress disorder, and affect distress: A review and neurocognitive model. Psychol. Bull. 2007; 133: 482–528. 17469988 10.1037/0033-2909.133.3.482

[3] Hulot J , Roseau J‐B , Gomez‐Merino D , Chennaoui M , Saguin E . Clinical description of sleep and trauma‐related nightmares in a population of French active‐duty members and veterans with post‐traumatic stress disorder. Encéphale 2024; 50: 11–19. 36424208 10.1016/j.encep.2022.10.002

[4] Phelps AJ , Forbes D , Creamer M . Understanding posttraumatic nightmares: An empirical and conceptual review. Clin. Psychol. Rev. 2008; 28: 338–355. 17629384 10.1016/j.cpr.2007.06.001

[5] Phelps AJ , Forbes D , Hopwood M , Creamer M . Trauma‐related dreams of Australian veterans with PTSD: Content, affect and phenomenology. Aust. N. Z. J. Psychiatry 2011; 45: 853–860. 21859279 10.3109/00048674.2011.599314

[6] Germain A . Sleep disturbances as the hallmark of PTSD: Where are we now? Am. J. Psychiatry 2013; 170: 372–382. 23223954 10.1176/appi.ajp.2012.12040432 PMC4197954

[7] Shore JH , Orton H , Manson SM . Trauma‐related nightmares among American Indian veterans: Views from the dream catcher. Am. Indian Alsk. Native Ment. Health Res. 2009; 16: 25–38. 19340764 10.5820/aian.1601.2009.25

[8] So CJ , Miller KE , Gehrman PR . Sleep disturbances associated with posttraumatic stress disorder. Psychiatr. Ann. 2023; 53: 491–495. 38293647 10.3928/00485713-20231012-01 PMC10825808

[9] Youngren WA , Hamilton NA , Preacher KJ . Assessing triggers of Posttrauma nightmares. J. Trauma. Stress 2020; 33: 511–520. 32521086 10.1002/jts.22532

[10] Saguin E , Feingold D , Sipahimalani G *et al*. PTSD symptom severity associated with sleep disturbances in military personnel: Evidence from a prospective controlled study with ecological recordings. Depress. Anxiety 2025; 2025: 8011375. 40370761 10.1155/da/8011375 PMC12077967

[11] Saguin E , Gomez‐Merino D , Sauvet F , Leger D , Chennaoui M . Sleep and PTSD in the military forces: A reciprocal relationship and a psychiatric approach. Brain Sci. 2021; 11: 1310. 34679375 10.3390/brainsci11101310 PMC8533994

[12] Pigeon WR , Campbell CE , Possemato K , Ouimette P . Longitudinal relationships of insomnia, nightmares, and PTSD severity in recent combat veterans. J. Psychosom. Res. 2013; 75: 546–550. 24290044 10.1016/j.jpsychores.2013.09.004

[13] Richards A , Santistevan A , Kovnick M *et al*. Distressing dreams in trauma survivors: Using a sleep diary mobile app to reveal distressing dream characteristics and their relationship to symptoms and suicidal ideation in trauma‐exposed adults. SLEEP Adv. 2025; 6: zpae099. 39896752 10.1093/sleepadvances/zpae099 PMC11786194

[14] Raskind MA , Peskind ER , Chow B *et al*. Trial of prazosin for post‐traumatic stress disorder in military veterans. N. Engl. J. Med. 2018; 378: 507–517. 29414272 10.1056/NEJMoa1507598

[15] Morgenthaler TI , Auerbach S , Casey KR *et al*. Position paper for the treatment of nightmare disorder in adults: An American Academy of sleep medicine position paper. J. Clin. Sleep Med. 2018; 14: 1041–1055. 29852917 10.5664/jcsm.7178 PMC5991964

[16] Raskind MA , Peterson K , Williams T *et al*. A trial of prazosin for combat trauma PTSD with nightmares in active‐duty soldiers returned from Iraq and Afghanistan. Am. J. Psychiatry 2013; 170: 1003–1010. 23846759 10.1176/appi.ajp.2013.12081133

[17] Saguin E , Feingold D , Roseau J‐B *et al*. An ecological approach to clinically assess nightmares in military service members with severe PTSD. Sleep Med. 2023; 103: 78–88. 36764045 10.1016/j.sleep.2023.01.024

[18] Garfinkel SN , Liberzon I . Neurobiology of PTSD: A review of neuroimaging findings. Psychiatr. Ann. 2009; 39: 370–381.

[19] Pole N . The psychophysiology of posttraumatic stress disorder: A meta‐analysis. Psychol. Bull. 2007; 133: 725–746. 17723027 10.1037/0033-2909.133.5.725

[20] Phelps AJ , Kanaan RAA , Worsnop C, Redston S , Ralph N , Forbes D . An ambulatory polysomnography study of the post‐traumatic nightmares of post‐traumatic stress disorder. Sleep 2018; 41: zsx188. 10.1093/sleep/zsx188 29182727

[21] Richards A , Woodward SH , Baquirin DPG *et al*. The sleep physiology of nightmares in veterans with psychological trauma: Evaluation of a dominant model using participant‐applied electroencephalography in the home environment. J. Sleep Res. 2023; 32: e13639. 10.1111/jsr.13639 PMC13054705 35644523

[22] Miller KE , Jamison AL , Gala S , Woodward SH . Two independent predictors of nightmares in posttraumatic stress disorder. J. Clin. Sleep Med. 2018; 14: 1921–1927. 30373691 10.5664/jcsm.7494 PMC6223551

[23] Hurwitz TD , Mahowald MW , Kuskowski M , Engdahl BE . Polysomnographic sleep is not clinically impaired in Vietnam combat veterans with chronic posttraumatic stress disorder. Biol. Psychiatry 1998; 44: 1066–1073. 9821572 10.1016/s0006-3223(98)00089-4

[24] Woodward SH , Arsenault NJ , Murray C , Bliwise DL . Laboratory sleep correlates of nightmare complaint in PTSD inpatients. Biol. Psychiatry 2000; 48: 1081–1087. 11094141 10.1016/s0006-3223(00)00917-3

[25] McCall CA , Watson NF . A narrative review of the association between post‐traumatic stress disorder and obstructive sleep apnea. J. Clin. Med. 2022; 11: 415. 35054110 10.3390/jcm11020415 PMC8780754

[26] Boucsein W . Electrodermal Activity, 2nd edn. Springer Science + Business Media, New York, NY, US, 2012.

[27] Dawson ME , Schell AM , Filion DL . The electrodermal system. In: Handbook of Psychophysiology, 4th edn. Cambridge University Press, New York, NY, 2017; 217–243.

[28] Maris E , Oostenveld R . Nonparametric statistical testing of EEG‐ and MEG‐data. J. Neurosci. Methods 2007; 164: 177–190. 17517438 10.1016/j.jneumeth.2007.03.024

[29] Saguin E , Hulot LJ , Roseau J‐B *et al*. Translation, cross‐cultural adaptation and preliminary validation of a French version of the trauma‐related nightmare survey (TRNS‐FR) in a PTSD veteran population. Mil. Med. 2023; 188: 3182–3190. 35472134 10.1093/milmed/usac107 PMC10464873

[30] Kobayashi I , Boarts JM , Delahanty DL . Polysomnographically measured sleep abnormalities in PTSD: a meta‐analytic review. Psychophysiology 2007; 44: 660–669. 17521374 10.1111/j.1469-8986.2007.537.x

[31] Thayer JF , Lane RD . Claude Bernard and the heart‐brain connection: Further elaboration of a model of neurovisceral integration. Neurosci. Biobehav. Rev. 2009; 33: 81–88. 18771686 10.1016/j.neubiorev.2008.08.004

[32] Thayer JF , Lane RD . A model of neurovisceral integration in emotion regulation and dysregulation. J. Affect. Disord. 2000; 61: 201–216. 11163422 10.1016/s0165-0327(00)00338-4

[33] Aston‐Jones G , Cohen JD . An integrative theory of locus coeruleus‐norepinephrine function: Adaptive gain and optimal performance. Annu. Rev. Neurosci. 2005; 28: 403–450. 16022602 10.1146/annurev.neuro.28.061604.135709

[34] Pitman RK , Rasmusson AM , Koenen KC *et al*. Biological studies of post‐traumatic stress disorder. Nat. Rev. Neurosci. 2012; 13: 769–787. 23047775 10.1038/nrn3339 PMC4951157

[35] Wiltshire CN , Wanna CP , Stenson AF *et al*. Associations between children's trauma‐related sequelae and skin conductance captured through mobile technology. Behav. Res. Ther. 2022; 150: 104036. 35078028 10.1016/j.brat.2022.104036 PMC8887191

[36] Hinrichs R , van Rooij SJ , Michopoulos V *et al*. Increased skin conductance response in the immediate aftermath of trauma predicts PTSD risk. Chronic Stress (Thousand Oaks) 2019; 3: 2470547019844441. 10.1177/2470547019844441 PMC6553652 31179413

[37] Siciliano RE , Anderson AS , Compas BE . Autonomic nervous system correlates of posttraumatic stress symptoms in youth: Meta‐analysis and qualitative review. Clin. Psychol. Rev. 2022; 92: 102125. 35078039 10.1016/j.cpr.2022.102125 PMC8858870

[38] Grasser LR , Saad B , Bazzi C *et al*. Skin conductance response to trauma interview as a candidate biomarker of trauma and related psychopathology in youth resettled as refugees. Eur. J. Psychotraumatol. 2022; 13: 2083375. 35713586 10.1080/20008198.2022.2083375 PMC9196716

[39] Mäder T , Oliver KI , Daffre C *et al*. Autonomic activity, posttraumatic and nontraumatic nightmares, and PTSD after trauma exposure. Psychol. Med. 2023; 53: 731–740. 34127168 10.1017/S0033291721002075 PMC9121310

[40] Kobayashi I , Lavela J , Mellman TA . Nocturnal autonomic balance and sleep in PTSD and resilience. J. Trauma. Stress. 2014; 27: 712–716. 25403523 10.1002/jts.21973

[41] Billman GE . The effect of heart rate on the heart rate variability response to autonomic interventions. Front. Physiol. 2013; 4: 222. 23986716 10.3389/fphys.2013.00222 PMC3752439

[42] Shaffer F , Ginsberg JP . An overview of heart rate variability metrics and norms. Front. Public Health 2017; 5: 258. 29034226 10.3389/fpubh.2017.00258 PMC5624990

[43] Craig ADB . How do you feel—now? The anterior insula and human awareness. Nat. Rev. Neurosci. 2009; 10: 59–70. 19096369 10.1038/nrn2555

[44] Paulus MP , Stein MB . Interoception in anxiety and depression. Brain Struct. Funct. 2010; 214: 451–463. 20490545 10.1007/s00429-010-0258-9 PMC2886901

[45] Nielsen T , Levin R . Nightmares: A new neurocognitive model. Sleep Med. Rev. 2007; 11: 295–310. 17498981 10.1016/j.smrv.2007.03.004

[46] Critchley HD . Electrodermal responses: What happens in the brain. Neuroscientist 2002; 8: 132–142. 11954558 10.1177/107385840200800209

[47] Lehrer PM , Gevirtz R . Heart rate variability biofeedback: How and why does it work? Front. Psychol. 2014; 5: 756. 25101026 10.3389/fpsyg.2014.00756 PMC4104929

[48] Woodward SH , Michell G , Santerre C . The psychophysiology of PTSD nightmares. In: Vermetten E , Germain A , Neylan TC (eds). Sleep and Combat‐Related Post Traumatic Stress Disorder. Springer, New York, NY, 2018 [Cited 2025 Sept 17]; 233–242.

[49] Woodward SH , Leskin GA , Sheikh JI . Movement during sleep: Associations with posttraumatic stress disorder, nightmares, and comorbid panic disorder. Sleep 2002; 25: 669–676. 12224848

[50] Mysliwiec V , Brock MS , Creamer JL , O'Reilly BM , Germain A , Roth BJ . Trauma associated sleep disorder: A parasomnia induced by trauma. Sleep Med. Rev. 2018; 37: 94–104. 28363448 10.1016/j.smrv.2017.01.004

[51] van der Kolk BA . The body keeps the score: Memory and the evolving psychobiology of posttraumatic stress. Harv. Rev. Psychiatry 1994; 1: 253–265. 9384857 10.3109/10673229409017088

[52] Remadi M , Dinis S , Bernard L *et al*. Evaluation of sleep and therapeutic education needs of military with PTSD. Encéphale 2024; 50: 48–53. 36907668 10.1016/j.encep.2023.01.004

