# Supplementary figures and images for: Physiological markers of Trauma‐related nightmares among military personnel suffering from PTSD: A multicenter home‐recording study
Source: Psychiatry Clin Neurosci. 2026 Feb 14;80(5):398–408. doi: 10.1111/pcn.70038 (PMC13139809; doi:10.1111/pcn.70038)

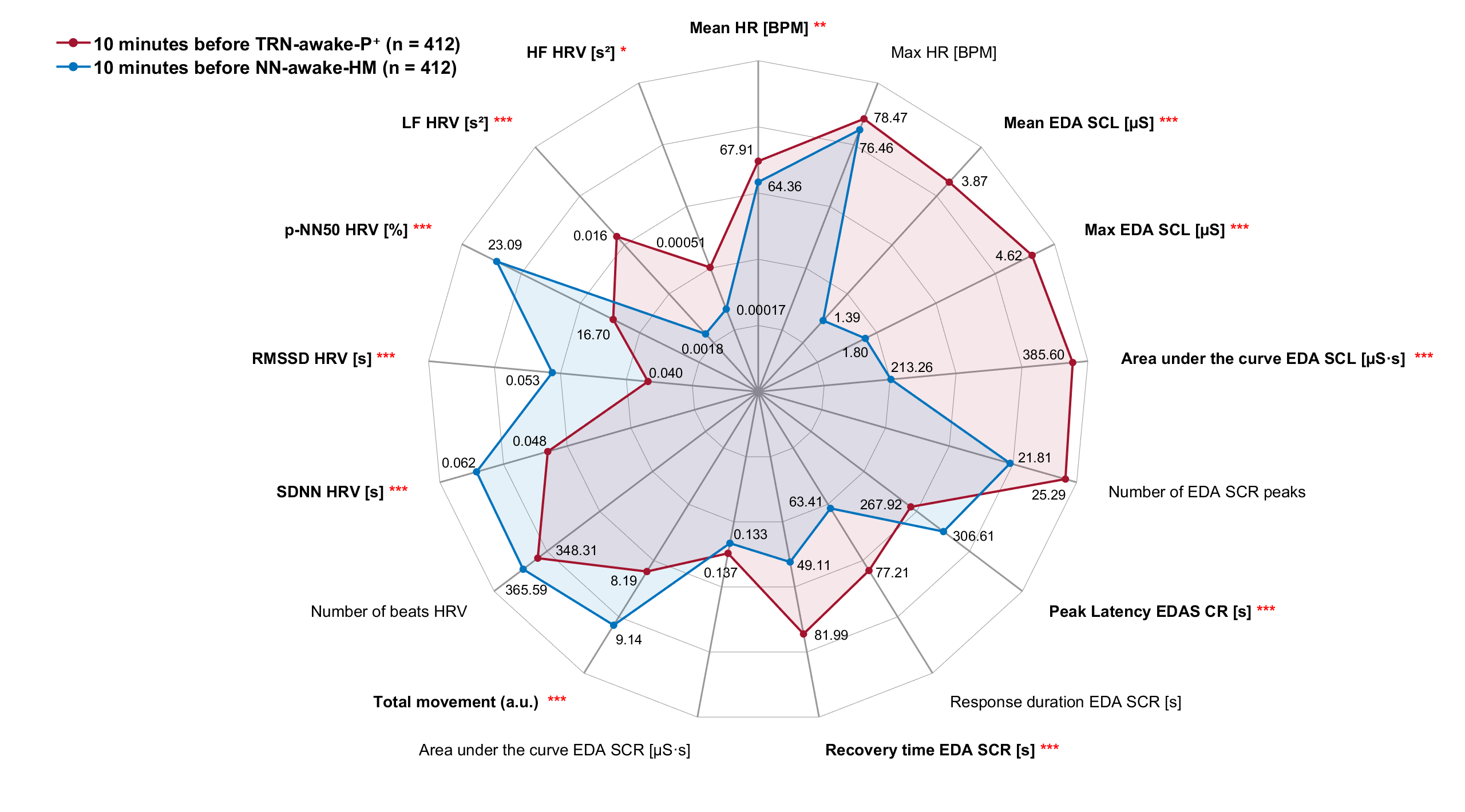

Supplement: Supplementary file 1 — Figure S1. Physiological signature preceding TRN‐awake‐P+ versus NN‐awake‐HM awakenings. Comparison of physiological profiles during the 10 min preceding trauma‐related nightmare (TRN)‐related awakenings in PTSD patients (TRN‐awake‐P+, N = 412, in red) and spontaneous awakenings in healthy military controls (NN‐awake‐HM, N = 412, in blue). Each axis of the spider plot represents a physiological variable measured in the 10 min before awakening: Heart rate (HR), Heart rate variability (HRV), tonic and phasic Electrodermal activity (EDA, SCL, and SCR), and body movement. HR reflects cardiac arousal; HRV indices capture parasympathetic and sympathetic balance; tonic and phasic EDA index sympathetic activation; and movement reflects motor activity surrounding awakening. Red asterisks indicate statistically significant differences between conditions (P <0.05, <0.01, <0.001), computed using Kruskal–Wallis tests with Bonferroni correction for multiple comparisons. Values plotted correspond to mean values per group. [file PCN-80-398-s001.tif]

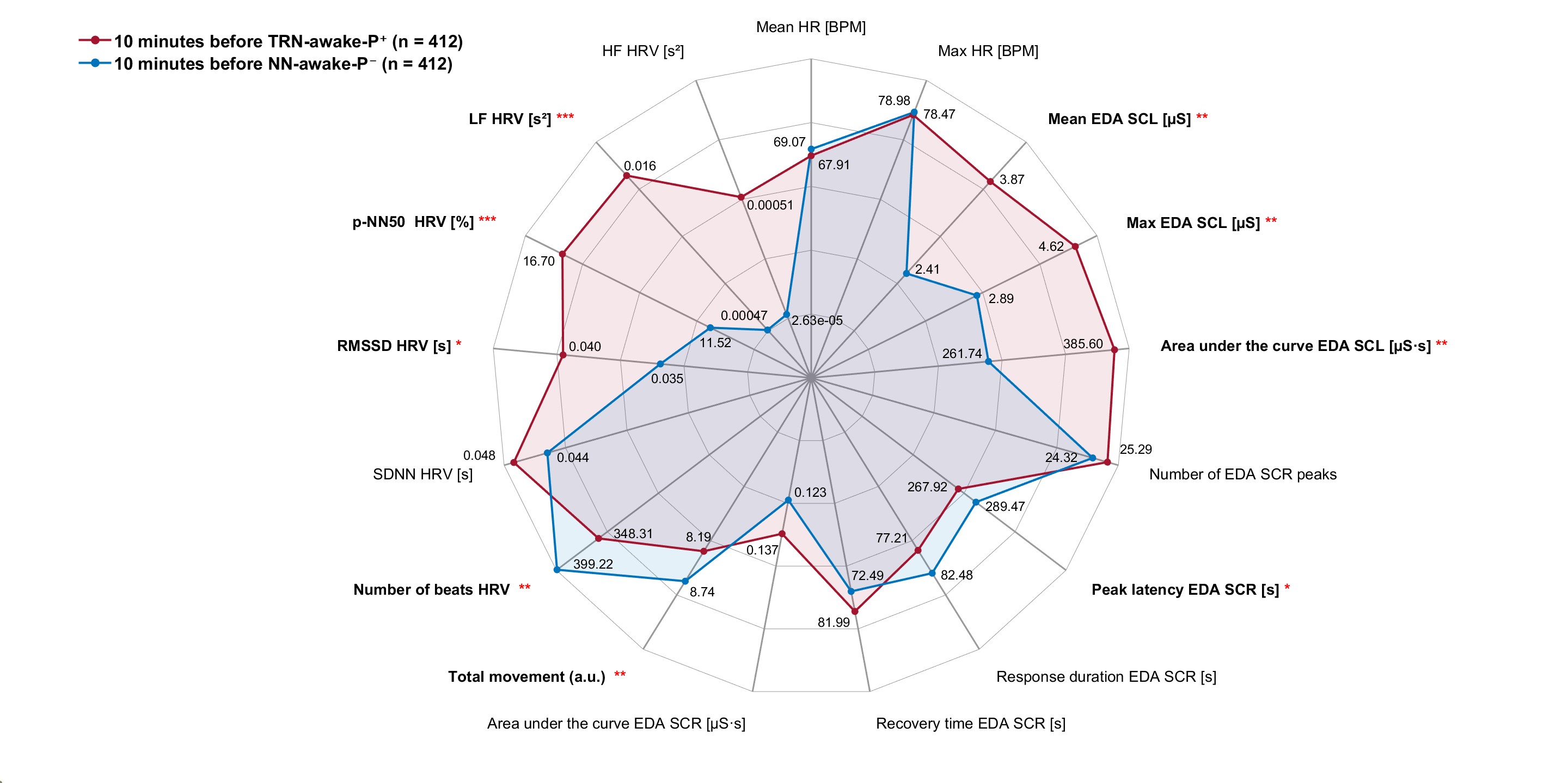

Supplement: Supplementary file 2 — Figure S2. Physiological signature preceding TRN‐awake‐P+ versus NN‐awake‐P− awakenings. Comparison of physiological profiles during the 10 min preceding trauma‐related nightmare (TRN)‐related awakenings in PTSD patients (TRN‐awake‐P+, N = 412, in red) and non‐nightmare awakenings in PTSD patients without any TRN awakenings (NN‐awake‐P−, N = 412, in blue). Each axis of the spider plot represents a physiological variable measured in the 10 min before awakening: Heart rate (HR), Heart rate variability (HRV), tonic and phasic Electrodermal activity (EDA, SCL, and SCR), and body movement. HR reflects cardiac arousal; HRV indices capture parasympathetic and sympathetic balance; tonic and phasic EDA index sympathetic activation; and movement reflects motor activity surrounding awakening. Red asterisks indicate statistically significant differences between conditions (P <0.05, <0.01, <0.001), computed using Kruskal–Wallis tests with Bonferroni correction for multiple comparisons. Values plotted correspond to mean values per group. [file PCN-80-398-s002.tif]
